# Correlates of sitting time in adults with type 2 diabetes

**DOI:** 10.1186/s12889-015-2086-6

**Published:** 2015-08-19

**Authors:** Anne-Sophie Brazeau, Samantha Hajna, Lawrence Joseph, Kaberi Dasgupta

**Affiliations:** Department of Medicine, McGill University, 687 Pine Avenue West, V-Building (V0.06), Montréal, QC H3A 1A1 Canada

**Keywords:** Sedentary behaviors, Seasons, Steps, Socio-demographic factors

## Abstract

**Background:**

Studies suggest a relationship between sitting time and cardiovascular disease mortality. Our aim was to identify socio-demographic, contextual, and clinical (e.g., body composition, diabetes duration) correlates of self-reported sitting time among adults with type 2 diabetes, a clinical population at high risk for cardiovascular disease. We sought to determine if there was an inverse relationship between sitting and step counts in a diabetes cohort in whom we had previously identified low step counts with further lowering in fall/winter.

**Methods:**

The cohort included 198 adults (54 % men; age 60.0 SD 11.5 years; Body mass index 30.4 SD 5.6 kg/m^2^) (Montréal, Canada). Socio-demographic, contextual and clinical factors were assessed using standardized questionnaires and step counts with a pedometer over 14 days (concealed viewing windows). Total sitting time was estimated once per season (up to 4 times per year at –month intervals) using the *International Physical Activity Questionnaire*-*Short version*. Potential sitting time correlates were evaluated using Bayesian longitudinal hierarchical linear regression models in participants with sitting time data (*n* = 191).

**Results:**

The average sitting time was 308 (SD 161) minutes/day without variation across seasons. Sitting time correlates were being an immigrant (56 fewer minutes/day spent sitting compared to non- immigrants, 95 % credible interval, CrI: −100, −11) and having a university degree (55 more minutes/day spent sitting compared to those without a university degree, 95 % CrI: 10, 100) after adjustment for potential correlates observed in univariate analyses (sex, age, job status, waist circumference, depressed mood, steps). Correlation between sitting and steps, adjusted for age and sex, was −0.144 (95 % CI: −0.280, 0.002).

**Conclusion:**

There was low correlation between sitting time and step counts. Therefore, high sitting time and low step counts are behaviours that may need to be independently targeted. Interventions to reduce sitting time in adults with type 2 diabetes may need to target non-immigrants and those with a university degree.

## Background

Greater use of modern technology and electronic entertainment has contributed to reductions in physical movement [1]. Adults with established type 2 diabetes are characterized by low levels of physical activity with step counts falling in the low active category according to the cut-offs proposed by Tudor-Locke and Bassett [2, 3]. We have previously determined pedometer-assessed daily step counts to average 5365 steps/day (Standard deviation, SD = 2655) in this clinical population, with higher step counts during the spring/summer seasons compared to the fall/winter seasons [4]. We have also determined higher step counts to be associated with lower blood pressure [5], A1C, adiposity measures [6], consistent with the impact of pedometer-based interventions [7] and a recent longitudinal analysis demonstrating the effects of higher step counts on reductions in cardiovascular mortality [8].

While increasing step counts is clearly an imperative in adults with type 2 diabetes, an area that is emerging as an additional issue is sitting time. Sedentary and sitting time have been linked not only with the development of type 2 diabetes and cardiovascular disease [9, 10], but also with both cardiovascular-specific and all-cause mortality (greatest compared to lowest sedentary time pooled hazard ratio 1.9, 95 % confidence interval (CI) 1.4, 2.7 [9] and >7 h/week compared to <1 h/day of television watching hazard ratio 1.5, 95 % CI 1.1, 2.0, [11]). Although there is between-country variation, average sitting time in adult populations, measured by self-report, is approximately 300 to 360 min per day (i.e., 5 to 6 h) [12, 13].

Both high sitting time and low step counts are thus undesirable behaviours for optimal health. It is not clear whether those with low step counts are the same group of individuals with high sitting time. Clarification of this issue would help to strengthen the developing of health behaviour-enhancing interventions aiming to reduce vascular disease risk in type 2 diabetes. To effectively address this newly-emerging vascular risk factor, it would be helpful to identify the sitting time predictors and correlates, particularly in groups at increased risk for mortality, such as people with type 2 diabetes [14].

Therefore, the aims of this study were, in adults with type 2 diabetes, 1) to quantify overall daily sitting time and variation across seasons, 2) to compare sitting and step counts in this clinical population, and 3) to identify the socio-demographic, contextual and health-related factors of self-reported sitting time in this population. We sought to determine if there was an inverse relationship between sitting and pedometer-assessed step counts and if there was seasonal variation in sitting time similar to that previously observed for step counts.

## Methods

This study is a secondary analysis of a prospective cohort study of adults (*n* = 201) with physician-diagnosed type 2 diabetes recruited through McGill University-affiliated outpatient clinics conducted in Montréal (Canada). Following informed consent, participants underwent consecutive seasonal assessments (i.e., 4 assessments) over a one-year follow-up period between June 2006 and June 2009. Complete description of the protocol can be found elsewhere but important elements are described herein [4, 15]. Procedures were approved by McGill University’s Faculty of Medicine Institutional Review Board and all participating institutions (McGill University Health Centre Research Ethics Board, Research Ethics Committee at the Jewish General Hospital and the Comité d'éthique de la recherche du Centre de santé et de services sociaux de la Montagne). The present analysis included data on 198 participants with at least one self-reported value for sitting (i.e., at one of the 4 assessments).

### Outcome measurement

Sitting time was assessed using the *International Physical Activity Questionnaire*-*Short version* (IPAQ-SV) (www.ipaq.ki.se) [16]. Participants were queried on the average time spent sitting during week days (hours and minutes per day, converted to minutes per day). This question included sitting time at work, at home, while doing course work and during leisure time. Examples were provided (e.g., time spent sitting at a desk, sitting to watch television). IPAQ-SV has acceptable reliability and validity for assessing usual sitting time [17, 18]. A recent validation study among 1751 adults demonstrated a correlation coefficient of 0.46 between the IPAQ-SV sitting question and 7-day accelerometry data (Actigraph GT1M) [19].

### Potential correlates examined

#### Step counts

Perception of walking was evaluated with the IPAQ-SV. Participants were asked 2 questions (during the last 7 days, on how many days did you walk for at least 10 min at a time and how much time did you usually spend walking on one of those days) and average walking time was computed according to the guidelines on how to process the data [20]. Objective measure of walking was assessed with pedometer counts. Step counts were quantified at each evaluation for a 2-week period [4, 15]. Participants were provided with three pedometers labelled A, B and C (Yamax SW-200, viewing window concealed). They wore one (pedometer A) for one week and a second (pedometer B) for another week during waking hours. They then mailed back all three to the study centre in the stamped and addressed envelope provided. The step counts recorded on the third pedometer (C) served to quantify the counts accumulated during the mailing process. This number was subtracted from the totals on the other two pedometers (A minus C and B minus C). The corrected step counts on pedometers A and B were summed and divided by the total number of days pedometers were worn as reported by the participants on a form included in the envelope.

#### Sociodemographic factors

Sex, education (university education, yes/no), annual household income (< $50,000, yes/no), work status (currently working, yes/no), marital status (married/common-law, yes/no), immigrant status (yes/no), and age (years) were assessed at baseline using a standardized questionnaire. For annual household income participants had to choose from 5 different categories (<$15,000; $15,000 to $30,000; $30,000 to $50,000; $50,000 to $80,000; > $80,000) and the two last were collapsed in the present analysis.

#### Contextual factors

In this analysis, season was based on corresponding solstice calendar definitions of fall (September 22/23 to December 20/21), winter (December 21/22 to March 19/20), spring (March 20/21 to June 19/20), and summer (June 20/21 to September 21/22) in the northern hemisphere. Dog ownership (yes/no) was queried as part of the baseline questionnaire. Vehicle access (yes/no) was assessed as part of a follow-up questionnaire that was mailed to all participants in the winter of 2012/2013.

#### Clinical measures

Depressed mood (yes/no) was assessed using the Center for Epidemiologic Studies-Depression Scale (CES-D Score ≥16), a validated tool for assessing depressive symptomology [21]. Body mass index (BMI in kg/m^2^) was calculated based on direct weight and height measurements and waist circumference (cm) was measured midway between the lateral lower ribs and the iliac crests. Insulin use (yes/no) and diabetes duration (years) were self-reported at the baseline visit [15].

### Statistical analysis

Data are presented as mean and SD unless state otherwise. Pearson correlations with 95 % CI were calculated. To compare the fall/winter and the spring/summer seasons, we averaged the data collected within these periods and computed absolute differences. Given the similarity of data from spring and summer and from fall and winter, spring and summer data were collapsed, as were fall and winter data, as in a previous analysis of this cohort [4]. Bayesian longitudinal hierarchical linear regression models with diffuse priors were used to estimate the associations of self-reported sitting time in minutes/day measured over time with step counts and each of the socio-demographic, contextual and clinical factors described above (WinBUGS 1.4.3). A series of models were unadjusted, partially adjusted and fully adjusted for the variables identified *a priori* as potential sitting time correlates. The fully adjusted final model was based on complete case data at baseline (*n* = 191) and included age, gender, immigrant status, education, waist circumference, job status, steps and absence of depressed mood. These determinants were selected given their important association with sitting time in univariate analyses. The importance of findings was based on examination of point estimates and 95 % credible intervals (95 % CrI), the Bayesian analog of frequentist confidence intervals.

## Results

**Fig. 1:**
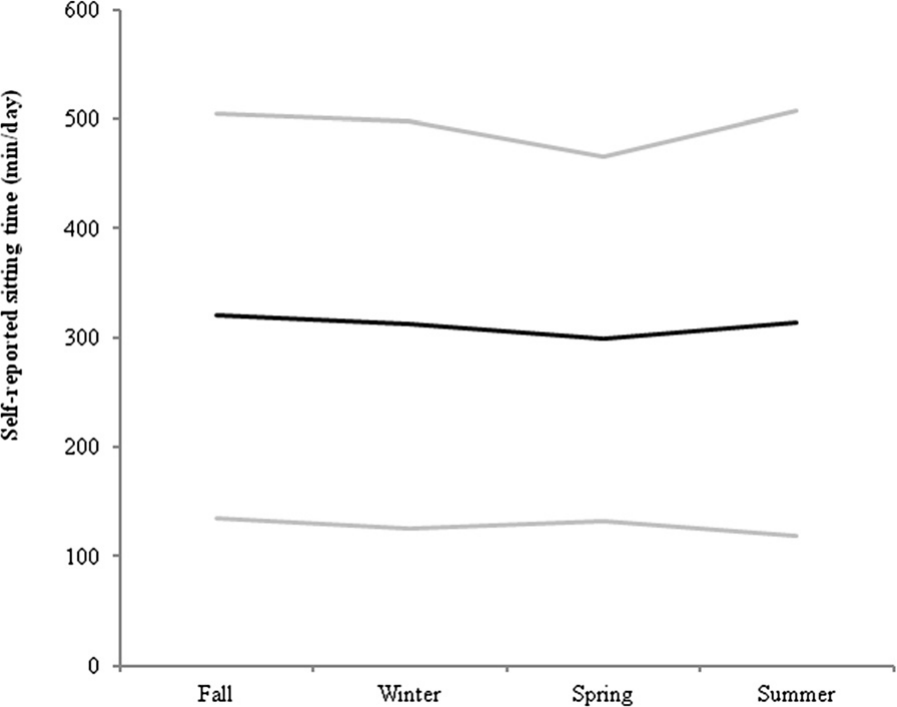
Self-reported sitting across seasons. Self-reported sitting: Fall, *n* = 154, Winter, *n* = 146; Spring, *n* = 150; Summer, *n* = 149. Data are mean values (*black line*) with 1 standard deviation (*grey lines*)

Average sitting time was 308 min/day (SD 161) equivalent to approximately five hours per day (Table 1). Fifty-one percent of the participants attended all 4 visits, 23.7 % attended 3 visits,13.6 % attended 2 visits and 11.6 % attended only1 visit. A plot of reported sitting time by season suggested no variation across seasons (Fig. 1).

**Table 1:** Univariate longitudinal hierarchical linear regression estimates between the predictors of interest and daily sitting time in minutes

		Baseline characteristics *N* = 198	Increment in minutes of daily sitting time (95 % CrI)^b^
Average daily sitting time (minutes);	*mean (SD)*	308 (161)	---
	*median (IQR)*	278 (188, 405)	---
Socio-demographic factors			
Men; *n (%)*		106 (54 %)	−2.9 (−4.9, −0.8)
Age (years); *mean (SD)*		60.0 (10.5)	41.3 (−3.5, 85.7)
University education; *n (%)*		78 (39 %)	58.2 (12.3, 103.6)
Currently employed; *n (%)*		113 (57 %)	86.3 (42.5, 130.0)
Annual household income ≥ $50,000; *n (%)* ^a^		77 (44 %)	90.0 (43.3, 134.9)
Married/common-law; *n (%)* ^a^		123 (69 %)	−34.9 (−86.3, 15.4)
Immigrant; *n (%)*		91 (46 %)	−53.3 (−98.2, −10.1)
Contextual factors			
Dog ownership; *n (%)*		31 (16 %)	18.2 (−40.9, 78.3)
Regular vehicle access; *n (%)* ^a^		70 (80 %)	92.0 (6.7, 179.2)
Steps (steps/day); *mean (SD)* ^a^		5361 (2473)	0.001 (−0.005, 0.008)
Clinical factors			
Body mass index (kg/m^2^); *mean (SD)*		30.4 (5.6)	1.3 (−2.8, 5.4)
Waist circumference (cm); *mean (SD)*		102.1 (13.2)	2.4 (1.1, 3.6)
Depressed mood; *n (%)*		55 (28 %)	−36.5 (−71.5, −2.4)
Diabetes duration (years); *mean (SD)*		9.4 (8.0)	−2.6 (−5.4, 0.2)
Insulin use; *n (%)*		66 (33 %)	---

^a^Annual household income (*n* = 177); married/common law (*n* = 178); regular vehicle access (*n* = 87); Daily steps (*n* = 129); Depressed mood (*n* = 137)

^b^Modelling independent variables at baseline except for depression and steps/day which were modelled over time; sitting time was modelled over time

**Fig. 2:**
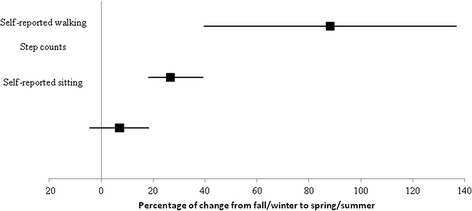
Behaviours’ change between seasons. Changes between seasons were available for 142 individuals. Data are mean with 95 % confidence intervals

Correlation between average self-reported sitting time and pedometer-assessed step counts, adjusted for age and sex, was −0.144 (95 % CI −0.280, 0.002). We observed a 26.6 % (95 % CI 18.2, 39.1) higher step count value during spring/summer but the spring/summer- fall/winter difference in sitting time was inconclusive (7.1 %; 95 % CI −4.4, 18.7) (Fig. 2).

Univariate associations with sitting time were observed for socio-demographic factors (age, immigrant status, university education, annual household income, job status) and for clinical factors (waist circumference and depressed mood) (Table 1). In the subgroup of participants with information on vehicle access (*n* = 87), having regular access to a vehicle was associated with almost a half-hour increase in daily sitting time (92.0 min/day, 95 % CrI 6.7, 179.2) compared to those not having access to a vehicle. Because BMI and waist circumference were strongly correlated (*r* = 0.83, 95 % CI 0.79, 0.87), only waist circumference was included in the final multivariate model. Vehicle access was not included in the final multivariate model because data were only available for a subsample. The impact of clinical factors decreased when integrated into a full multivariate model. In the multivariate model, being an immigrant was associated with less sitting time (−55.7, 95 % CrI: −99.9, −11.3) and having completed a university degree was associated with more (54.8, 95 % CrI: 10.0, 100.3) (Table 2).

**Table 2:** Multivariate longitudinal hierarchical linear regression estimates between the predictors of interest and daily sitting time in minutes (*n* = 191)

	Increment in minutes of daily sitting time (95 % CrI)
Sex (men)	22.5 (−23.3, 68.3)
Age, per year	−2.4 (−5.0, 0.0)
Currently working	42.9 (−9.1, 93.4)
University education	54.8 (10.0, 100.3)
Immigrant	−55.7 (−99.9, −11.3)
Waist circumference, per cm	1.1 (−0.7, 2.9)
Absence of depressed mood	−11.1 (−61.2, 38.6)
Daily steps, per step	−0.001 (−0.011, 0.008)

## Discussion

In our study sample of adults with type 2 diabetes, an average of over 5 h of sitting time/day was reported. While university-educated individuals reported sitting 55 min/day more than other individuals, immigrants reported 56 min/day less than non-immigrants. There was no seasonal variation in sitting time observed. Further, there was no important association between self-reported sitting time and pedometer-assessed step counts. Therefore, strategies aimed at reducing sitting time appear to require targeting a subgroup that differs somewhat from those with lower step counts. The absence of association differed from a previous study among young adult workers among whom there was a negative association between self-reported sitting and walking [22].

Studies in other groups (e.g. Canadian Community Health Survey, Epic Norfolk) [23, 24] have largely focused on television and screen viewing as metrics of sedentary time. Older age, being unemployed or retired, higher body mass index and depressive symptomology have been shown to be associated with more television viewing [25]. However, increased sitting time may be related to work and transport-related factors, not just television viewing; less is known about overall sitting time and its correlates [25]. The amount of sitting time reported in our type 2 diabetes cohort is similar to that reported in population-based multi-country studies using self-reported measures [12, 13]. We further assessed for differences across seasons. In contrast to step counts (758 fewer steps/day in fall/winter compared to spring/summer) [4], we demonstrated that sitting time did not vary importantly across seasons. Thus, the seasonal reduction in steps does not appear to directly lead to an increase in overall sitting time. One possibility that we considered was that the absence of seasonal variation in sitting time was the result of using self-report methods. However, with respect to physical activity, our analyses demonstrated seasonal variation not only for objectively-assessed step counts but also for self-reported walking, as captured through the IPAQ-SV. Both measures of walking showed important increases during spring/summer compared to fall/winter.

Accordingly, correlates of reported sitting time differ from those associated with steps [26, 27]. Indeed, it has been demonstrated that interventions aimed at increasing physical activity do not necessarily reduce sitting time [28]. A recent systematic review also reported a weak to moderate inverse relationship between sedentary behaviors in general and physical activity [29]. Further, even among those achieving the recommended 150 min/week of physical activity, sitting more than 8 h/day still confers an elevated risk of all-cause mortality [30]. Thus, it appears that both low levels of physical inactivity and high amounts of sitting time need to be separately targeted to achieve maximum health benefit [31].

Given that the IPAQ-SV queries overall sitting time on weekdays, a large part of self-reported sitting time may be work-related. High educational level is often associated with professional office setting environments (i.e., so-called “white-collar” jobs). These are in contrast to “blue-collar” jobs that are generally more physically demanding. The higher time spent sitting observed among people with a university degree may thus reflect the type of job they have. Consistent with our findings, low education status was negatively related to self-reported weekday sitting time among young adult Australian women (unstandardized B −1.23, 95 % CI −1.38, −1.09) [32]. Similarly, in this Australian study, people with a blue-collar occupation and those working less than 35 h per week reported sitting less during the week [32]. However, given that 43 % of our cohort was retired or unemployed, having a university degree in some individuals was likely related to non work-related activities that increased sitting time.

Immigrants demonstrated lower sitting time, even after adjustment for work status, educational level and age. Recent immigrants are twice as likely as Canadian-born Montréal residents to use public transit in their daily commute [33]. Compared to Canadian born individuals, immigrants are more likely to work in processing and manufacturing industries and sales and service occupations [34]. These factors may in part explain the lower daily sitting time observed in this group of individuals.

Although having a car was associated with increased sitting time (92.0 min/day, 95 % CrI 6.7, 179.2) in univariate analysis, we did not include this variable in the multivariate analysis because data were available for only 44 % of our sample. This one and half hour increase in self-reported sitting time in this subgroup is nonetheless substantial. In addition to university education and not being an immigrant, in univariate analyses, younger age, currently working, high annual income, depressed mood, and higher waist circumference were all associated with higher sitting time; further, male sex, not being in a married or common-law relationship, and shorter diabetes duration showed trends towards associations with more sitting time. In multivariate analyses, associations of sitting time with university education and not being an immigrant were confirmed. There were trends suggestive of associations with younger age and currently working, but these were not conclusive.

The IPAQ is the most widely used instrument to assess sitting at the population level and thus its use facilitates comparison with other studies. We acknowledge that the IPAQ-SV does not distinguish between the different domains of sitting, such as work-related, transportation, home-related, and leisure-time nor does it provide information on extended sitting time versus sitting occurring in short bouts. Moreover it queries only the information about week days in contrast to the IPAQ long version. Both the short and long versions are subject to recall bias in contrast to objective accelerometry measures. Healy and colleagues, in a review of validation studies, reported low-to-moderate correlations between self-reported sitting and accelerometer-assessed sedentary time. The IPAQ-SV tends to underestimate sitting time when compared to accelerometer-assessed sedentary time [35]. While this may partly be the result of recall bias, it may also be due to the fact that IPAQ specifically queries sitting, a subset of sedentary behaviour, while accelerometry captures overall sedentary behaviors and not specifically sitting. The French version of the IPAQ long form has been validated in a cohort of diabetes patients (*n* = 143; 60.9 SD 10.5 years of age) and showed similar results to non-clinical populations [36]. Despite the limitations of using the IPAQ-SV in our study, it does allow us to specifically focus on the issue of sitting and its distinction from low step counts in type 2 diabetes.

## Conclusion

Our analyses did not demonstrate a relationship between sitting time and clinical variables; however, there is evidence from other larger studies of such a relationship [9, 10]. In our study, adults with type 2 diabetes reported an average sitting time similar to the general population that was without variation across seasons. Socio-demographic factors, such as having completed a university degree and being a non-immigrant, were linked to self-reported sitting time. There was no correlation between sitting time and step counts. Therefore, high sitting time and low step counts are behaviours that may need to be independently targeted. Interventions to reduce sitting time could include home and work environments designed to allow tasks to be completing from a standing position (e.g., standing desks, standing-only office meetings/gatherings or standing up every hour to break prolonged sitting) [37].

## Abbreviations

BMI: Body mass index
CES-D: Center for Epidemiologic Studies – Depression scale
CI: Confidence interval
CrI: Credible interval
IPAQ-SV: International Physical Activity Questionnaire – Short version
IQR: Interquartile range
SD: Standard deviation
